# Factors associated with successful discontinuation of certolizumab pegol in early rheumatoid arthritis

**DOI:** 10.1111/1756-185X.13780

**Published:** 2020-01-19

**Authors:** Yoshiya Tanaka, Tatsuya Atsumi, Kazuhiko Yamamoto, Tsutomu Takeuchi, Hisashi Yamanaka, Naoki Ishiguro, Katsumi Eguchi, Akira Watanabe, Hideki Origasa, Toshiharu Shoji, Nobuyuki Miyasaka, Takao Koike

**Affiliations:** ^1^ The First Department of Internal Medicine University of Occupational and Environmental Health, Japan Kitakyushu Japan; ^2^ Hokkaido University Graduate School of Medicine Sapporo Japan; ^3^ Center for Integrative Medical Sciences RIKEN Kanagawa Japan; ^4^ Keio University School of Medicine Tokyo Japan; ^5^ Institute of Rheumatology Tokyo Women's Medical University Tokyo Japan; ^6^ Faculty of Medicine Nagoya University Graduate School Nagoya Japan; ^7^ Rheumatic and Collagen Disease Center Sasebo Chuo Hospital Sasebo Japan; ^8^ Tohoku Bunka Gakuen University Sendai Japan; ^9^ Division of Biostatistics and Clinical Epidemiology The University of Toyama Graduate School of Medicine Toyama Japan; ^10^ UCB Pharma Tokyo Japan; ^11^ Tokyo Medical and Dental University Tokyo Japan; ^12^ Hokkaido Medical Center for Rheumatic Diseases Sapporo Japan

**Keywords:** anti‐TNF, certolizumab pegol, discontinuation, early rheumatoid arthritis, prognostic factor

## Abstract

**Aim:**

The Certolizumab–Optimal Prevention of joint damage for Early Rheumatoid Arthritis (C‐OPERA) study demonstrated that in methotrexate (MTX)‐naïve early RA patients with poor prognostic factors, 1‐year certolizumab pegol (CZP) therapy added to the first year of 2‐year optimized MTX therapy brings radiographic and clinical benefits through 2 years even after stopping CZP. This exploratory analysis aimed to identify factors at baseline and at CZP discontinuation associated with successful CZP discontinuation.

**Methods:**

MTX‐naïve early RA patients with poor prognostic factors entered C‐OPERA (NCT01451203), a multicenter, randomized controlled trial. Patients were randomized to CZP + MTX (n = 159) or PBO + MTX (n = 157); those who completed the 1‐year, double‐blind period received MTX alone in Year 2 (CZP + MTX→MTX, n = 108; PBO + MTX→MTX, n = 71). Association between factors at baseline or at discontinuation of CZP and clinical/radiographic outcomes were evaluated by multiple logistic regression analysis. Predictive value cut‐offs were calculated using receiver operating characteristic analysis.

**Results:**

Sex (male) and low baseline Disease Activity Score of 28 joints – erythrocyte sedimentation rate (DAS28‐ESR) were associated with simple disease activity index (SDAI) remission (≤3.3), whereas high baseline DAS28‐ESR and modified total Sharp score (mTSS) were associated with clinically relevant radiographic progression (yearly progression mTSS > 3) at Week 104 (across both treatment arms). Low DAS28‐ESR (<2.1) and rheumatoid factor (RF; <74 IU/mL) at discontinuation of CZP were associated with SDAI remission at Week 104. At Week 104, SDAI remission was achieved by 75.0% (42/56) of patients with low DAS28‐ESR and RF at discontinuation, compared to 15.4% (2/13) of patients with high DAS28‐ESR and RF.

**Conclusion:**

Patients with low RF and low disease activity after treatment with CZP + MTX may be able to discontinue CZP without risk of loss of response.

## 1 INTRODUCTION

Rheumatoid arthritis (RA) is a chronic inflammatory disease characterized by progressive inflammatory synovitis, joint destruction and chronic disability. Anti‐tumor necrosis factor (anti‐TNF) agents provide an effective treatment option for patients with RA.^1^, ^2^ Certolizumab pegol (CZP) is a humanized anti‐TNF antibody fragment conjugated to polyethylene glycol, approved for the treatment of inflammatory diseases, including RA.^3^ Early treatment of patients with CZP has been associated with a reduction in inflammation, inhibition of structural damage and improved long‐term outcomes.^4^, ^5^ Recent studies have investigated whether the clinical benefits of early CZP treatment are maintained following CZP withdrawal.

The Certolizumab–Optimal Prevention of joint damage for Early RA (C‐OPERA) study was a phase 3, 2‐year, multicenter study.^5^, ^6^ C‐OPERA demonstrated the clinical benefit of initial 1‐year CZP treatment on 2‐year optimized methotrexate (MTX) therapy, in MTX‐naïve early RA patients with poor prognostic factors. C‐OPERA was double‐blind (DB) for the first year, with patients randomized to receive either CZP or placebo (PBO) alongside MTX.^6^ For the second year (post‐treatment [PT] period), CZP and PBO were discontinued and all patients were maintained on MTX alone.^5^ During the PT period, although most patients who had received CZP + MTX maintained good clinical status after withdrawal of CZP, approximately 25% of patients (28/108) went on to flare.^5^ Identifying factors at baseline or at CZP discontinuation that are associated with disease flare may help clinicians decide whether to cease or continue CZP treatment after attaining the desired response with initial CZP + MTX treatment.

In this present study, we performed post‐hoc analyses on the C‐OPERA data to identify factors at baseline and at CZP discontinuation associated with successful CZP discontinuation.

## 2 METHODS

In the C‐OPERA study (NCT01451203), the efficacy and safety of CZP + MTX were compared with PBO + MTX in MTX‐naïve patients with early RA in Japan. Details of the C‐OPERA study have been published elsewhere.^5^, ^6^ In brief, eligible patients were within 1 year of disease onset and had poor prognostic factors including: positive for anti‐cyclic citrullinated peptide antibody (anti‐CCP, >3 times the upper limit of the normal range [ULN]), positive for rheumatoid factor (RF) and/or bone erosions on radiographs of the hands or feet. Prior to the DB period, patients were randomized to receive either PBO + MTX or CZP + MTX. Optimized oral MTX was started at 8 mg/wk and escalated up to 16 mg/wk by Week 8, if tolerated. Patients who completed the 52‐week DB period were eligible to enter the 52‐week PT period. In the PT period, administration of PBO or CZP was discontinued but patients remained on optimized MTX therapy. Disease activity and van der Heijde modified total Sharp score (mTSS) were evaluated at baseline, Week 52, Week 104, and at withdrawal. Last observation carried forward analysis was employed for clinical outcomes, and linear extrapolation was used for estimation of mTSS in patients who withdrew from the study.

### 2.1 Baseline factors associated with simple disease activity index (SDAI) remission and clinically relevant radiographic progression at Week 52 in PBO + MTX patients

Associations between baseline factors and Week 52 outcomes were analyzed by logistic regression analysis. Factors included in the analysis were: Disease Activity Index of 28 joints – erythrocyte sedimentation rate (DAS28‐ESR), Health Assessment Questionnaire Disability Index (HAQ‐DI), mTSS, RF, anti‐CCP antibody, matrix metalloproteinase (MMP)‐3, tumor necrosis factor (TNF)α, and interleukin (IL)‐6. Log value + 1 was used to normalize mTSS, RF, anti‐CCP antibody, MMP‐3, TNFα, and IL‐6 values. Univariate logistic regression analysis was used to identify factors that were possibly associated with SDAI remission (<3.3) and clinically relevant radiographic progression (cRRP, yearly progression mTSS > 3, unpublished). Multivariate logistic regression analysis was applied for factors with a *P* value <0.1 to identify independent predictive factors. Factors with a *P* value <.05 were considered to be associated.

### 2.2 Baseline factors associated with maintenance of clinical response

Analyses in this section were performed for all patients who entered the C‐OPERA study (irrespective of treatment arm allocation). The association of baseline factors and CZP treatment with Week 104 outcomes was evaluated by logistic regression analysis. Patient characteristics, including age, gender, and body mass index (BMI), and baseline disease status, including DAS28‐ESR, HAQ‐DI, mTSS, RF, anti‐CCP antibody, and MMP‐3 were evaluated in the analysis. Log value + 1 was used to normalize mTSS, RF, anti‐CCP antibody and MMP‐3 values. Multiple logistic regression analysis was applied to factors identified with a *P* value <.1 during the univariate analysis.

### 2.3 Factors at CZP discontinuation associated with maintenance of clinical response

All analyses in this section were performed for patients who were originally allocated to the CZP + MTX group and entered the PT period. Association between factors at Week 52 (discontinuation of CZP) and Week 104 outcomes were analyzed by logistic regression analysis. Factors included in the analysis were: DAS28‐ESR, HAQ‐DI, mTSS, RF, anti‐CCP antibody, and MMP‐3. Log value + 1 was used for mTSS, RF, anti‐CCP antibody, and MMP‐3 to normalize values. Univariate logistic regression analysis was used to identify factors associated with the clinical outcomes. Factors with *P* value <.1 were included in the multiple logistic regression analysis to identify independent predictive factor(s); the Youden index on the receiver operating characteristic curves was used to estimate appropriate cut‐off values for these factors. The proportion of patients achieving SDAI remission was calculated for patients with values that were lower than, and equal to or higher than, the cut‐off value of the relevant predictive factor(s).

## 3 RESULTS

Patients in the C‐OPERA study were randomized to CZP + MTX (n = 159) or PBO + MTX (n = 157); patients who completed the 1‐year, DB period received MTX alone in Year 2 (CZP + MTX→MTX, n = 108; PBO + MTX→MTX, n = 71).^5^, ^6^ As previously reported, SDAI remission (≤3.3) at baseline, Week 52, and Week 104 was 0.0% (0/157), 33.8% (53/157) and 29.3% (46/157) in the PBO + MTX group, and 0.0% (0/159), 57.9% (92/159) and 41.5% (66/159) in the CZP + MTX group, respectively.^5^, ^6^ In patients who were treated with CZP + MTX in Year 1 (DB period) and entered Year 2 (PT period), the previously reported SDAI remission at Week 52 and Week 104 was 79.6% (86/108) and 55.6% (60/108), respectively.^5^

cRRP during Year 1 (DB period) was 15.3% (24/157) and 5.0% (8/159), in PBO + MTX and CZP + MTX, respectively. cRRP during Year 2 (PT period) in patients who were treated with CZP + MTX in Year 1 was 0.9% (1/108).

### 3.1 Baseline factors associated with a clinical response at Week 52 in the PBO + MTX treatment group

In the MTX + PBO arm, low baseline DAS28‐ESR and low HAQ‐DI were associated with SDAI remission at Week 52 by univariate analysis (Table 1). After multivariate adjustment, low DAS28‐ESR was the only factor associated with SDAI remission (odds ratio 0.57, 95% CI 0.38‐0.86, *P* = .007). Using univariate analysis, baseline DAS28‐ESR, HAQ‐DI, mTSS, MMP‐3, and IL‐6 were all shown to associate with cRRP in the PBO + MTX group. Multivariate analyses identified mTSS as the only independent predictive factor for cRRP (Table 1).

**Table 1:** Logistic regression analysis of association between baseline factors and clinical and radiographic outcomes at Week 52 in patients assigned to the PB0 + MTX treatment arm

	SDAI remission Odds ratio 95% CI *P* value		cRRP Odds ratio 95% CI *P* value	
	Univariate	Multivariate	Univariate	Multivariate
DAS28‐ESR	0.56 0.41‐0.77 <.001	**0.57** **0.38‐0.86** **.007**	2.03 1.33‐3.1 .001	1.39 0.74‐2.59 .302
HAQ‐DI	0.52 0.31‐0.88 .016	0.94 0.48‐1.83 .847	2.49 1.33‐4.65 .004	1.38 0.57‐3.35 .475
mTSS	0.82 0.59‐1.14 .229	‐	2.04 1.38‐3.02 <.001	**2.02** **1.3‐3.17** **.002**
RF	1.06 0.77‐1.45 .728	‐	1.51 0.97‐2.36 .068	1.18 0.74‐1.87 .492
Anti‐CCP antibody	0.88 0.59‐1.3 .509	‐	0.94 0.56‐1.56 .801	‐
MMP‐3	0.89 0.63‐1.27 .530	‐	1.82 1.14‐2.9 .012	1.00 0.54‐1.86 .989
TNFα	1.0 0.69‐1.65 .778	‐	1.16 0.69‐1.95 .576	‐
IL‐6	0.8 0.61‐1.05 .109	‐	1.98 1.32‐2.96 <.001	1.61 0.95‐2.72 .077

Abbreviations: CCP, cyclic citrullinated peptide; cRRP, clinically relevant radiographic progression; CZP, certolizumab pegol; DAS28‐ESR, Disease Activity Score of 28 joints ‐ erythrocyte sedimentation rate; HAQ‐DI, Health Assessment Questionnaire Disability Index; IL‐6, interleukin 6; MMP‐3, matrix metalloproteinase; mTSS, van der Heijde modified total Sharp score; MTX, methotrexate; PBO, placebo; RF, rheumatoid factor; SDAI, simple disease activity index; TNFα, tumor necrosis factor α.

### 3.2 Baseline factors associated with maintenance of clinical response

In univariate analyses, CZP treatment was associated with a higher SDAI remission rate and a lower cRRP rate at Week 104. Out of the remaining factors included in the analysis, only gender, BMI, baseline DAS28‐ESR and HAQ‐DI were also associated with SDAI remission at Week 104 (Table 2). After adjustment for these factors, Week 104 SDAI remission was associated with gender (*P* = .026), baseline DAS28‐ESR (*P* = .012), and CZP treatment (*P* = .047; Table 2).

**Table 2:** Logistic regression analysis of association between baseline factors and clinical and radiographic outcomes at Week 104 in patients randomized to CZP + MTX (n = 159) or PBO + MTX (n = 157)

	SDAI remission odds ratio (95% CI) *P* value		cRRP odds ratio (95% CI) *P* value	
	Univariate	Multiple	Univariate	Multiple
Age	0.99 (0.97‐1.01) .322		1.00 (0.96‐1.04) .950	
Gender	0.51 (0.29‐0.91) .022	**0.51** **(0.28‐0.92)** **.026**	1.20 (0.44‐3.26) .727	
BMI	0.94 (0.88‐1.01) .082	0.93 (0.87‐1.00) .051	0.97 (0.87‐1.08) .589	
DAS28‐ESR	0.63 (0.51‐0.79) <.001	**0.70** **(0.53‐0.92)** **.012**	2.03 (1.42‐2.90) <.001	**1.63** **(1.01‐2.62)** **.044**
HAQ‐DI	0.56 (0.38‐0.80) .002	0.78 (0.48‐1.26) .309	2.32 (1.34‐4.02) .003	1.37 (0.67‐2.81) .393
mTSS	0.77 (0.61‐0.98) .032	0.80 (0.62‐1.02) .077	2.08 (1.50‐2.89) <.001	**2.00** **(1.37‐2.92)** **<.001**
RF	1.00 (1.00‐1.00) .971		1.00 (1.00‐1.00) .009	1.00 (1.00‐1.00) .353
Anti‐CCP antibody	1.00 (1.00‐1.00) .381		1.00 (1.00‐1.01) .379	
MMP‐3	1.00 (1.00‐1.00) .312		1.00 (1.00‐1.00) .024	1.00 (1.00‐1.00) .948
CZP treatment	1.71 (1.07‐2.73) .024	**1.65** **(1.01‐2.69)** **.047**	0.33 (0.14‐0.76) .009	**0.33** **(0.13‐0.81)** **.017**

Abbreviations: BMI, body mass index; CCP, cyclic citrullinated peptide; cRRP, clinically relevant radiographic progression; CZP, certolizumab pegol; DAS28‐ESR, Disease Activity Score of 28 joints ‐ erythrocyte sedimentation rate; HAQ‐DI, Health Assessment Questionnaire Disability Index; MMP‐3, matrix metalloproteinase; mTSS, van der Heijde modified total Sharp score; MTX, methotrexate; PBO, placebo; RF, rheumatoid factor; SDAI, simple disease activity index.

Bold indicates factors shown to reach significance in univariate/multivariate analyses.

Baseline factors associated with cRRP were DAS28‐ESR, HAQ‐DI, mTSS, RF, MMP‐3, and CZP treatment. After adjustment for these factors, Week 104 cRRP was associated with baseline DAS28‐ESR (*P* = .044), mTSS (*P* < .001), and CZP treatment (*P* = .017; Table 1).

### 3.3 Factors at CZP discontinuation associated with maintenance of clinical response

**Figure 1:**
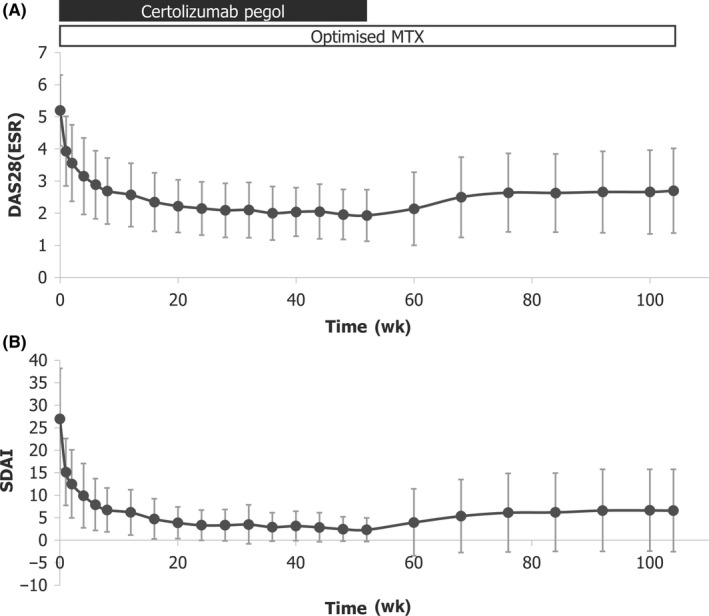
Change in disease activity in patients who were treated with CZP + MTX in Year 1 and entered the post‐treatment period (CZP + MTX→MTX; N = 108). CZP, certolizumab pegol; DAS28(ESR), disease activity score 28(erythrocyte sedimentation rate); MTX, methotrexate; SDAI, Simple Disease Activity Index

**Figure 2:**
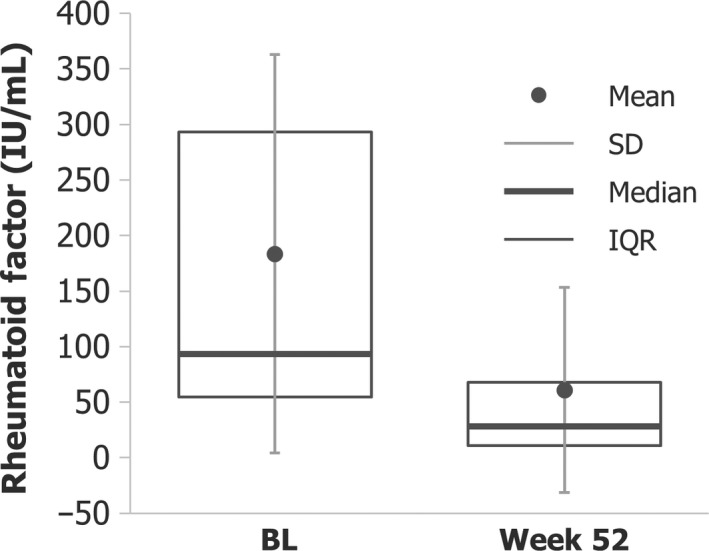
Reduction in rheumatoid factor levels in patients who were treated with CZP + MTX in Year 1 and entered the post‐treatment period (CZP + MTX→MTX; N = 108). BL, baseline; CZP, certolizumab pegol; IQR, inter‐quartile range; MTX, methotrexate

Univariate and multiple logistic regression analyses showed that DAS28‐ESR and RF at CZP discontinuation (Week 52) were associated with SDAI remission 1 year after stopping CZP treatment (Week 104; Table 3). There was a rapid reduction in disease activity (indicated by both DAS28‐ESR and SDAI measures) with CZP treatment, and only a slight increase was observed after discontinuation of CZP (Figure 1). RF at discontinuation of CZP treatment (Week 52) was lower compared to baseline (Figure 2).

**Table 3:** Logistic regression analysis of association between factors at Week 52 (CZP discontinuation) and clinical outcomes at Week 104 in patients who were treated with CZP + MTX in Year 1 and entered the post‐treatment period (CZP + MTX→MTX, n = 108)

	SDAI remission odds ratio (95% CI) *P* value	
	Univariate	Multiple
DAS28‐ESR	0.29 (0.16‐0.54) <.001	**0.29** **(0.16‐0.54)** **<.001**
HAQ‐DI	0.47 (0.11‐2.03) .309	
mTSS	0.85 (0.57‐1.26) .408	
RF	0.69 (0.50‐0.95) .022	**0.67** **(0.47‐0.96)** **.028**
Anti‐CCP antibody	0.86 (0.63‐1.19) .370	
MMP‐3	0.77 (0.35‐1.70) .516	

Abbreviations: CCP, cyclic citrullinated peptide; CZP, certolizumab pegol; DAS28‐ESR, Disease Activity Score of 28 joints ‐ erythrocyte sedimentation rate; HAQ‐DI, Health Assessment Questionnaire Disability Index; MMP‐3, matrix metalloproteinase; mTSS, van der Heijde modified total Sharp score; MTX, methotrexate; RF, rheumatoid factor; SDAI, simple disease activity index.

**Figure 3:**
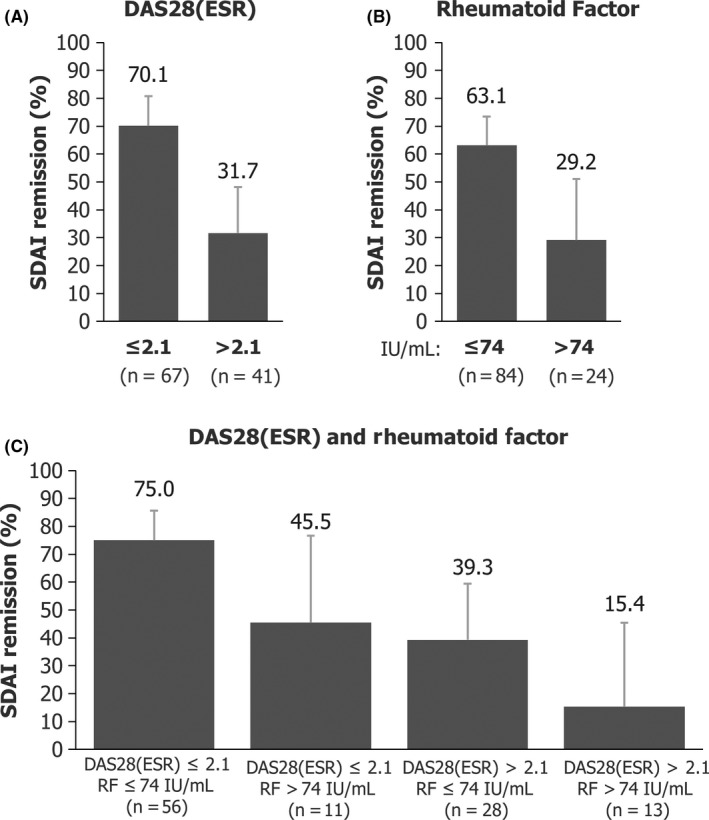
Subgroup analyses of SDAI remission at Week 104 by DAS28(ESR) and RF at Week 52 in patients who were treated with CZP + MTX in Year 1 and entered the post‐treatment period (CZP + MTX→MTX; N = 108). CZP, certolizumab pegol; DAS28(ESR), disease activity score 28(erythrocyte sedimentation rate); MTX, methotrexate; RF, rheumatoid factor; SDAI, Simple Disease Activity Index

**Figure 4:**
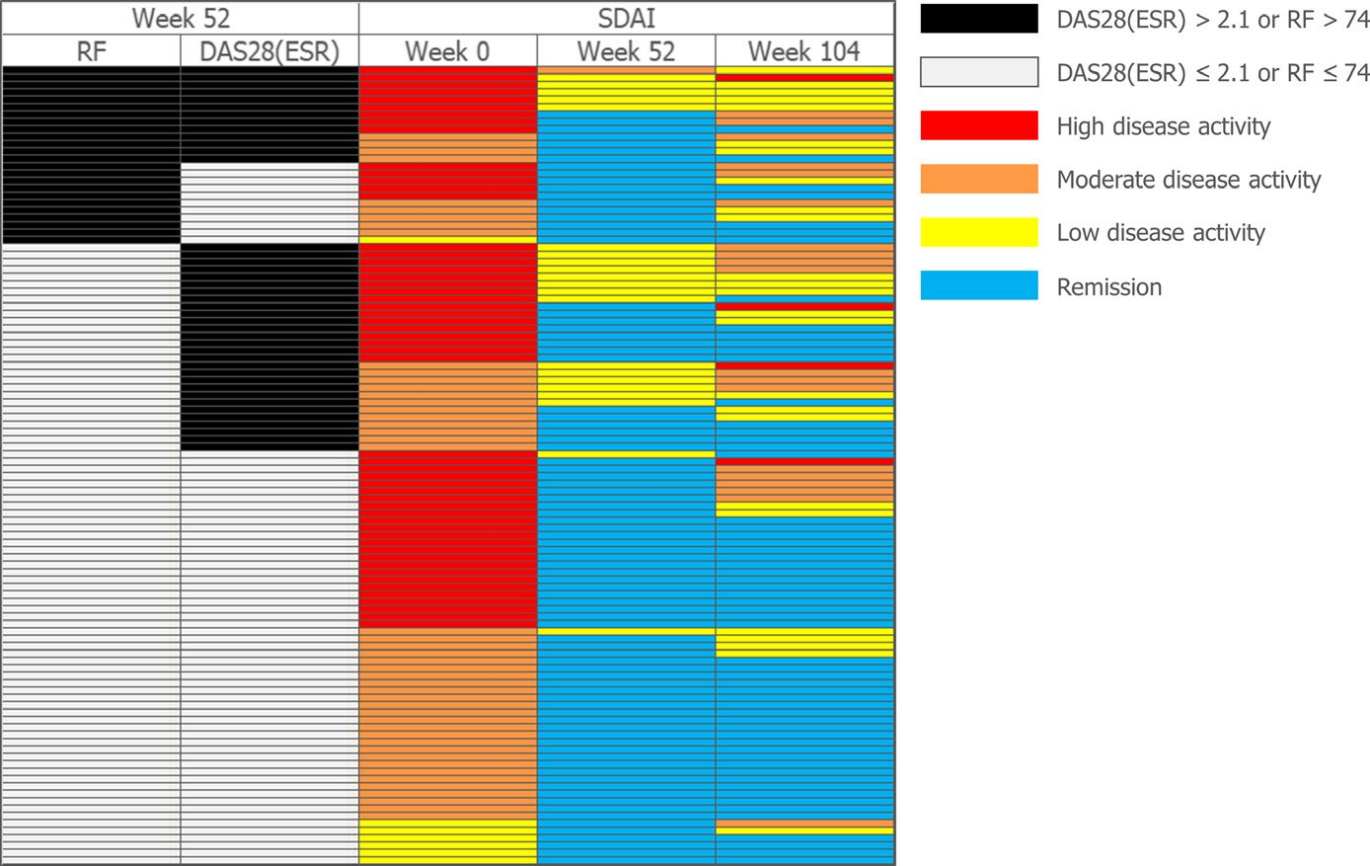
Distribution of disease activity by Week 52 DAS28(ESR) and rheumatoid factor in patients who were treated with CZP + MTX in Year 1 and entered the post‐treatment period (CZP + MTX→MTX; N = 108). CZP, certolizumab pegol; DAS28(ESR), disease activity score 28(erythrocyte sedimentation rate); MTX, methotrexate; RF, rheumatoid factor; SDAI, Simple Disease Activity Index

Calculated Week 52 DAS28‐ESR and RF cut‐off values for SDAI remission at Week 104 were 2.1 and 74 IU/mL, respectively. SDAI remission rates at Week 104 in patients with lower and higher DAS28‐ESR at discontinuation were 70.1% (47/67) and 31.7% (13/41), respectively. Out of the patients with lower RF values at discontinuation of CZP, 63.1% (53/84) achieved SDAI remission at Week 104, compared to 29.2% (7/24) of patients with RF values above the cut‐off (74 IU/mL) at discontinuation. Higher rates of SDAI remission (75.0%, 42/56) at Week 104 were observed in patients with lower DAS28‐ESR and RF at Week 52, whereas a very low remission rate (15.4%, 2/13) was reported among patients with higher values for both DAS28‐ESR and RF. Patients with higher DAS28‐ESR and lower RF, and patients with lower DAS28‐ESR and higher RF at Week 52 showed intermediate rates of remission (Figure 3). The distribution of disease activity at baseline, Week 52, and Week 104 stratified by Week 52 DAS28‐ESR and RF is presented in Figure 4. In patients with high disease activity at baseline, 16 out of 24 patients (66.7%) with low DAS28‐ESR and low RF at Week 52 were in SDAI remission at Week 104; whereas only 1 patient out of 9 (11.1%) with high DAS28‐ESR and high RF at baseline was in remission at Week 52.

## 4 DISCUSSION

In recent years, the use of MTX as an anchor drug, the introduction of biological disease‐modifying anti‐rheumatic drugs and implementation of treat‐to‐target (T2T) strategies, along with the introduction and increased use of early intervention treatment protocols, have markedly improved RA patient outcomes. Owing to these advances in RA treatment, many patients achieve either remission or a low disease activity state. Nevertheless, the number of patients with an active disease state is not negligible. Consequently, identification of risk factors associated with poor outcomes after initiation of treatment is important for the development of effective personalized treatment strategies.

Some studies have suggested the possibility of discontinuing or interrupting treatment with biologics after attainment of clinical response.^7^, ^8^, ^9^ However, identifying the patients who can discontinue treatment without going on to flare, can prove challenging. Therefore, it is of therapeutic value to identify factors that can be used to predict maintenance of a “good disease state” following discontinuation of treatment with biologics. Furthermore, if biologic treatment could be stopped successfully in some patients, it would dramatically reduce the economic burden of RA therapy and any risk of adverse events related to prolonged biologic use.

The post‐hoc analyses presented here aimed to provide clinicians with information to aid their decision‐making both at the start of treatment, to help determine whether to opt for biologic co‐therapy with MTX, and following disease control, to guide their decision regarding the discontinuation of biologic treatment.

### 4.1 Baseline factors associated with maintenance of clinical response

In C‐OPERA, a substantial number of patients withdrew from the study, particularly from the placebo group. In the post‐hoc analyses reported here, patient characteristics and baseline disease status were associated with clinical and radiographic outcomes; therefore, patient withdrawal due to disease activity has the potential to bias the observed results. However, after adjustment by multiple logistic regression analyses, there were significant improvements following treatment with CZP compared with placebo, suggesting that any bias had a limited impact on the results. Out of the factors evaluated, including patient characteristics and baseline disease status, sex (male) and a low DAS28‐ESR were identified as independent predictive factors of Week 104 SDAI remission, and high DAS28‐ESR and mTSS at baseline were associated with cRRP at Week 104. Although these findings should be interpreted with caution due to the high number of patient dropouts, these results are in line with findings from previous studies.^10^, ^11^, ^12^ Other factors, including auto‐antibodies, were not associated with Week 104 outcomes (*P* > .05). In the C‐OPERA study, only patients with high levels of anti‐CCP antibody at baseline were included, therefore, it is not possible to draw conclusions regarding the importance of baseline auto‐antibodies, especially anti‐CCP antibodies, in the prediction of long‐term outcomes.

### 4.2 Factors at CZP discontinuation associated with maintenance of clinical response

It has been suggested that discontinuation of biologic treatment without subsequent disease flare may be an achievable target, for example in early RA patients.^13^ In a previous study in early RA, lower disease activity at withdrawal (deep remission) was associated with discontinuation without disease flare,^7^, ^8^ and the importance of the immunological state of patients in achieving disease “cure” was emphasized.^14^

In C‐OPERA, patients treated with CZP + MTX who entered the PT period (discontinued CZP at Week 52) achieved SDAI remission rates at Weeks 52 and 104 of 79.6% and 55.6%, respectively.^5^ The post‐hoc analysis presented here included factors utilized in the assessment of disease status. Lower levels of disease activity and lower levels of RF were independently associated with higher remission rates after discontinuation of CZP. A better prediction of clinical response maintenance was observed when using a combination of DAS28‐ESR and RF, compared to DAS28‐ESR or RF alone. Patients with both low disease activity and low RF at CZP discontinuation had a lower risk of active disease, whereas patients with relatively high disease activity and high RF at Week 52 had a higher risk of loss of response at Week 104. A small number of patients had active disease despite low disease activity and low RF at discontinuation, which may suggest the involvement of other, unknown factors. Nevertheless, the data presented here implicate disease activity and RF in the maintenance of disease control after discontinuation of CZP treatment.

In CZP‐treated patients, both DAS28‐ESR and RF were reduced at Week 52 compared to baseline. Both DAS28‐ESR at baseline and at discontinuation were associated with SDAI remission at Week 104, indicating the importance of disease activity both before and after treatment with CZP. Overall, there was no association between baseline RF and Week 104 outcomes. This may reflect the greater importance of immunological status at discontinuation, versus at baseline before biologic initiation, in maintenance of remission after cessation of CZP treatment. Recently, factors associated with maintenance of low disease activity after stopping adalimumab in early RA patients were reported. In that report, disease activity and RF were associated with low disease activity 2 years after discontinuation of adalimumab.^15^ Taken together, immunological status PT and the good clinical status achieved with anti‐TNFs may play a crucial role in long‐term disease management in addition to the short‐term control of disease activity.

CZP + MTX patients who entered the PT period of the C‐OPERA study, maintained a very high radiographic non‐progression rate, despite some patients withdrawing from the study due to disease flare. Radiographic non‐progression during the first year of treatment was 91.7% and was maintained after CZP discontinuation (94.4%).^5^ Due to the small number of patients with radiographic progression in this group, the association between factors at Week 52 and cRRP was not analyzed.

The results of these post‐hoc analyses suggest that patients with low disease activity and low RF may be able to consider discontinuation of CZP, whereas patients with high disease activity and/or high RF should consider continuing CZP treatment.

## 5 CONCLUSIONS

The results of this post‐hoc analyses indicate that disease activity and joint damage at the start of treatment are predictive of long‐term outcomes in patients treated with optimized MTX plus CZP. Therefore, patients with poor prognostic factors may benefit from initial aggressive treatment with CZP and MTX. In addition to deep remission, low RF at discontinuation may be predictive of remission maintenance following discontinuation of CZP.

## CONFLICT OF INTEREST

YT: Research grants and/or consulting fees: AbbVie, Asahi Kasei, Astellas, AstraZeneca, Bristol‐Myers Squibb, Chugai, Daiichi‐Sankyo, Eisai, Eli Lilly, GlaxoSmithKline, Janssen, Mitsubishi Tanabe Pharma, MSD, Pfizer, Quintiles, Takeda, and UCB Pharma; TA: Speakers’ fees: Astellas, Bristol‐Myers Squibb, Chugai, and Mitsubishi Tanabe Pharma; KY: Research grants and/or consulting fees: Abbott, Bristol‐Myers Squibb, Chugai, Eisai, Mitsubishi Tanabe Pharma, Pfizer, Roche, Santen, and UCB Pharma; TT: Research grants: Astellas, Chugai, Daiichi‐Sankyo, Takeda, AbbVie GK, Asahi Kasei, Mitsubishi Tanabe Pharma, Pfizer Japan, Eisai, AYUMI, Nippon Kayaku, and Novartis; Speakers’ fees: AbbVie GK, Bristol‐Myers KK, Chugai, Mitsubishi Tanabe Pharma, Pfizer Japan, Astellas, Daiichi‐Sankyo, Eisai, Sanofi KK, Teijin Pharma, Takeda, Novartis KK; Consultant fees: AstraZeneca KK, Eli Lilly KK, Novartis KK, Mitsubishi Tanabe Pharma, AbbVie GK, Nippon Kayaku, Janssen KK, Astellas, Taiho Pharmaceutical, Chugai, Taisho Toyama Pharmaceutical, GlaxoSmithKline KK, and UCB Pharma Japan; HY: Research grants and/or consulting fees: Abbott, Astellas, Bristol‐Myers Squibb, Chugai, Eisai, Janssen, Mitsubishi Tanabe Pharma, Pfizer, Takeda, and UCB Pharma; NI: Research grants and/or speakers’ fees: Abbott, Astellas, Bristol‐Myers Squibb, Chugai, Eisai, Janssen, Kaken, Mitsubishi Tanabe Pharma, Otsuka, Pfizer, Taisho‐Toyama, and Takeda; KE: Consulting fees from UCB Pharma; AW: Research grants and/or speakers’ fees: Daiichi‐Sankyo, Dainippon‑Sumitomo, GlaxoSmithKline, Kyorin, Meiji Seika, Mitsubishi Tanabe Pharma, MSD, Pfizer, Shionogi, Taiho, and Taisho‐Toyama; HO: None declared; NM: None declared; TK: Speakers’ bureau: AbbVie, Astellas, Asuka Pharma, Bristol‑Myers, Chugai, Daiichi‐Sankyo, Eisai, Mitsubishi Tanabe Pharma, Pfizer Japan, Teijin Pharma, and UCB Pharma; Consultant for: Bristol‐Myers, Eli Lilly Japan, and Pfizer Japan.

## Data Availability

The datasets generated and analyzed for this article were derived from study data available in anonymized format upon reasonable request via the Clinical Study Data Request platform (http://www.clinicalstudydatarequest.com).
